# Editorial: Quorum-sensing in Gram-positive pathogens – mechanisms, role in infection, and potential as a therapeutic target

**DOI:** 10.3389/fcimb.2023.1236705

**Published:** 2023-06-19

**Authors:** Michael Otto, Seth W. Dickey, Christiane Wolz

**Affiliations:** ^1^ Pathogen Molecular Genetics Section, Laboratory of Bacteriology, National Institute of Allergy and Infectious Diseases, U.S. National Institutes of Health, Bethesda, MD, United States; ^2^ Department of Veterinary Medicine, University of Maryland, College Park, MD, United States; ^3^ Virginia-Maryland College of Veterinary Medicine, College Park, MD, United States; ^4^ Interfaculty Institute of Microbiology and Infection Medicine, University of Tübingen, Tübingen, Germany; ^5^ Cluster of Excellence EXC 2124 “Controlling Microbes to Fight Infections”, University of Tübingen, Tübingen, Germany

**Keywords:** quorum-sensing (QS), staphylococcus, streptococcus, AGR, infection, antivirulence

The term “quorum-sensing” (QS) describes a mechanism by which bacteria sense the density of the population and adjust gene expression accordingly. This mechanism allows the bacteria to adapt their physiology to the change in environmental conditions that accompanies the growth of the population, most notably a scarcity of nutrients (Miller and Bassler, 2001). During infection, the responsibility of QS control is believed to postpone the production of “public goods” until the bacteria have grown to growth-limiting densities, at which point the QS-controlled products can be produced at sufficient concentrations. Many of the QS-controlled factors are toxins and immune-modulatory molecules which for example control infiltrating immune cells (Le and Otto, 2015). At high density, the bacteria can also afford to produce QS-controlled degradative exoenzymes to acquire nutrients or essential ions from the host tissue.

QS is generally based on the secretion of a signal molecule, sometimes called pheromone or autoinducer (AI), which controls its own biosynthesis in a positive feedback loop and upon reaching a certain extracellular threshold concentration triggers a signal cascade that ultimately leads to the changes in the expression of QS-controlled genes (the QS regulon) (Miller and Bassler, 2001). Because accumulation of the signal requires a closed system and a threshold can also be reached with a relatively low number of bacteria in a small system with limited diffusion, QS has also been called “diffusion sensing” (Redfield, 2002).

QS in Gram-negative and Gram-positive bacteria generally follows the same general principle. However, in Gram-negative bacteria the QS systems use small, membrane-diffusible signal molecules, whereas Gram-positive bacteria use non-membrane diffusible peptide-based signals, sometimes called autoinducing peptides (AIPs) (Lyon and Novick, 2004). AIPs require dedicated export systems and membrane-located sensing systems or, in some cases, dedicated importers together with intracellular sensors. Membrane-located sensors commonly belong to the family of so-called two-component systems, which comprise a signal-binding membrane-spanning histidine kinase enzyme and a cognate cytoplasmic response regulator to which activation is transferred *via* phosphorylation (Miller and Bassler, 2001; Lyon and Novick, 2004).

Much of the considerable interest in QS stems from the fact that it controls virulence mechanisms and is thus a premier target for antivirulence drug development approaches (Dickey et al., 2017). Among QS systems of Gram-positive bacteria, the Agr system of *Staphylococcus aureus* has been most studied in terms of mechanism, control of virulence, and exploitation as an antivirulence target. The interesting phenomenon of species/subgroup-specific AIP variation and cross-inhibition that is present in this genus is being exploited as a basis for QS-targeted antivirulence approaches (Le and Otto, 2015).

Two papers in this series address QS-controlled virulence. Kinney et al. studied *S. aureus*-induced endocarditis by investigating in a rabbit model of endocarditis how the temporal expression of several global regulators of *S. aureus*, including Agr, correlates with signs of infection. They found that low expression of SarA and Agr is associated with vegetation formation as a hallmark of infective endocarditis. This is reminiscent of the notion of increased infectivity of *agr* mutants in other biofilm-associated infections such as prosthetic joint infection, cystic fibrosis, or device-associated bacteremia (Fowler et al., 2004; Goerke and Wolz, 2010; He et al., 2022).

Wang et al. focused on sepsis and found that the host response to Gram-negative and Gram-positive bacteria can vary considerably. These findings are in line with the recently developing idea that species-specific bacterial virulence factors, including those controlled by *S. aureus* Agr, have a key role in determining the outcome of sepsis (Cheung et al., 2021).

Recent findings also indicate Agr plays a key role for staphylococcal colonization and bacterial competition during colonization, which may be exploited for decolonization strategies (Piewngam et al., 2018; Nakamura et al., 2020). Tamai et al. in their review discuss Agr with a focus on its role in skin colonization and atopic dermatitis as well as a target for antivirulence drug development, a subject which has recently been pointed out requires more dedication, particularly using *in-vivo* infection models (Otto, 2023).

Some other Gram-positive bacteria also use Agr homologues (e.g., *Clostridioides difficile*, *Enterococcus faecalis*) but in these organisms, Agr control is not yet completely understood.

Most Gram-positive bacteria use QS systems that are not phylogenetically related to Agr, but they always also use peptide signals. Some Gram-positive bacteria have several QS systems, some of which may have very specific tasks in controlling a small and defined set of target genes. For example, group A streptococci control virulence, biofilm formation, and competence *via* the QS system Rgg, invasion-related genes by the *sil* locus, and synthesis of specific lantibiotic bacteriocins by yet further QS-like systems (Jimenez and Federle, 2014).

In addition to genus- or species-specific systems, there is a system called LuxS/AI-2 that has first been described in some Gram-negative bacteria and has been claimed to be “universal” (Schauder et al., 2001). AI-2 is a byproduct of the activated methyl cycle, a part of a housekeeping metabolism pathway that is conserved in bacteria. Whether it has a QS function in Gram-positive bacteria, or other bacteria in which no apparent AI-2 sensor is present, is controversial (Rezzonico and Duffy, 2008). Agnew et al. in their contribution provide evidence for a role of interaction between LuxS and a type 1 restriction-modification system in *Streptococcus pneumoniae*, which the authors’ results suggest may play a role in infection and niche adaptation.

While QS is a large field with many important studies being published almost constantly, we still hope that the studies published in this series will give readers some valuable additional insight especially given that QS in Gram-positive bacteria remains understudied in comparison.

## Author contributions

All authors listed have made a substantial, direct, and intellectual contribution to the work and approved it for publication.
